# Supplementary material for: Pyruvate Dehydrogenase Kinase 1 inhibition mediated oxidative phosphorylation enhancement in cartilage promotes osteoarthritis progression
Source: BMC Musculoskelet Disord. 2023 Jul 20;24:597. doi: 10.1186/s12891-023-06585-6 (PMC10357736; doi:10.1186/s12891-023-06585-6)
Supplement: Supplementary file 1 — Additional file 1. [file 12891_2023_6585_MOESM1_ESM.zip › KEGG.Gsea.1653622432464/KEGG_RIG_I_LIKE_RECEPTOR_SIGNALING_PATHWAY.html]

Details for gene set KEGG\_RIG\_I\_LIKE\_RECEPTOR\_SIGNALING\_PATHWAY[GSEA]

|  || Dataset | 55457\_collapsed\_to\_symbols.cls.cls #OA\_versus\_normal.cls.cls #OA\_versus\_normal\_repos |
| Phenotype | cls.cls#OA\_versus\_normal\_repos |
| Upregulated in class | normal |
| GeneSet | KEGG\_RIG\_I\_LIKE\_RECEPTOR\_SIGNALING\_PATHWAY |
| Enrichment Score (ES) | -0.3724608 |
| Normalized Enrichment Score (NES) | -1.4875165 |
| Nominal p-value | 0.02377179 |
| FDR q-value | 0.19714622 |
| FWER p-Value | 0.937 |
Table: GSEA Results Summary

  

Fig 1: Enrichment plot: KEGG\_RIG\_I\_LIKE\_RECEPTOR\_SIGNALING\_PATHWAY      
 Profile of the Running ES Score & Positions of GeneSet Members on the Rank Ordered List

  

| SYMBOL | TITLE | RANK IN GENE LIST | RANK METRIC SCORE | RUNNING ES | CORE ENRICHMENT || 1 | TANK | TRAF family member associated NFKB activator [Source:HGNC Symbol;Acc:HGNC:11562] | 439 | 0.690 | -0.0003 | No |
| 2 | MAPK9 | mitogen-activated protein kinase 9 [Source:HGNC Symbol;Acc:HGNC:6886] | 721 | 0.588 | 0.0068 | No |
| 3 | FADD | Fas associated via death domain [Source:HGNC Symbol;Acc:HGNC:3573] | 924 | 0.534 | 0.0173 | No |
| 4 | ATG5 | autophagy related 5 [Source:HGNC Symbol;Acc:HGNC:589] | 1142 | 0.488 | 0.0244 | No |
| 5 | IFNA8 | interferon alpha 8 [Source:HGNC Symbol;Acc:HGNC:5429] | 1206 | 0.475 | 0.0428 | No |
| 6 | MAP3K7 | mitogen-activated protein kinase kinase kinase 7 [Source:HGNC Symbol;Acc:HGNC:6859] | 1701 | 0.399 | 0.0240 | No |
| 7 | CXCL10 | C-X-C motif chemokine ligand 10 [Source:HGNC Symbol;Acc:HGNC:10637] | 1709 | 0.396 | 0.0429 | No |
| 8 | TNF | tumor necrosis factor [Source:HGNC Symbol;Acc:HGNC:11892] | 2098 | 0.350 | 0.0299 | No |
| 9 | IFIH1 | interferon induced with helicase C domain 1 [Source:HGNC Symbol;Acc:HGNC:18873] | 2204 | 0.338 | 0.0383 | No |
| 10 | PIN1 | "peptidylprolyl cis/trans isomerase, NIMA-interacting 1 [Source:HGNC Symbol;Acc:HGNC:8988]" | 2257 | 0.331 | 0.0506 | No |
| 11 | MAPK14 | mitogen-activated protein kinase 14 [Source:HGNC Symbol;Acc:HGNC:6876] | 2352 | 0.320 | 0.0590 | No |
| 12 | MAPK13 | mitogen-activated protein kinase 13 [Source:HGNC Symbol;Acc:HGNC:6875] | 2544 | 0.298 | 0.0588 | No |
| 13 | ISG15 | ISG15 ubiquitin like modifier [Source:HGNC Symbol;Acc:HGNC:4053] | 2821 | 0.268 | 0.0504 | No |
| 14 | RELA | "RELA proto-oncogene, NF-kB subunit [Source:HGNC Symbol;Acc:HGNC:9955]" | 3942 | 0.161 | -0.0289 | No |
| 15 | DHX58 | DExH-box helicase 58 [Source:HGNC Symbol;Acc:HGNC:29517] | 4130 | 0.143 | -0.0364 | No |
| 16 | IFNA4 | interferon alpha 4 [Source:HGNC Symbol;Acc:HGNC:5425] | 4282 | 0.129 | -0.0418 | No |
| 17 | IRF7 | interferon regulatory factor 7 [Source:HGNC Symbol;Acc:HGNC:6122] | 4411 | 0.120 | -0.0459 | No |
| 18 | NFKB1 | nuclear factor kappa B subunit 1 [Source:HGNC Symbol;Acc:HGNC:7794] | 4626 | 0.102 | -0.0575 | No |
| 19 | NLRX1 | NLR family member X1 [Source:HGNC Symbol;Acc:HGNC:29890] | 4696 | 0.096 | -0.0582 | No |
| 20 | TKFC | triokinase and FMN cyclase [Source:HGNC Symbol;Acc:HGNC:24552] | 4989 | 0.074 | -0.0773 | No |
| 21 | CYLD | CYLD lysine 63 deubiquitinase [Source:HGNC Symbol;Acc:HGNC:2584] | 5664 | 0.018 | -0.1289 | No |
| 22 | TRADD | TNFRSF1A associated via death domain [Source:HGNC Symbol;Acc:HGNC:12030] | 5713 | 0.015 | -0.1319 | No |
| 23 | TRAF6 | TNF receptor associated factor 6 [Source:HGNC Symbol;Acc:HGNC:12036] | 5761 | 0.011 | -0.1350 | No |
| 24 | IFNA2 | interferon alpha 2 [Source:HGNC Symbol;Acc:HGNC:5423] | 5869 | 0.004 | -0.1432 | No |
| 25 | IKBKB | inhibitor of nuclear factor kappa B kinase subunit beta [Source:HGNC Symbol;Acc:HGNC:5960] | 5914 | 0.001 | -0.1465 | No |
| 26 | IL12B | interleukin 12B [Source:HGNC Symbol;Acc:HGNC:5970] | 5991 | -0.004 | -0.1523 | No |
| 27 | RNF125 | ring finger protein 125 [Source:HGNC Symbol;Acc:HGNC:21150] | 6049 | -0.008 | -0.1563 | No |
| 28 | TRAF3 | TNF receptor associated factor 3 [Source:HGNC Symbol;Acc:HGNC:12033] | 6108 | -0.013 | -0.1602 | No |
| 29 | IFNA21 | interferon alpha 21 [Source:HGNC Symbol;Acc:HGNC:5424] | 6127 | -0.015 | -0.1609 | No |
| 30 | DDX58 | DExD/H-box helicase 58 [Source:HGNC Symbol;Acc:HGNC:19102] | 6480 | -0.041 | -0.1863 | No |
| 31 | IFNA10 | interferon alpha 10 [Source:HGNC Symbol;Acc:HGNC:5418] | 6682 | -0.054 | -0.1992 | No |
| 32 | IFNA5 | interferon alpha 5 [Source:HGNC Symbol;Acc:HGNC:5426] | 6755 | -0.059 | -0.2019 | No |
| 33 | IL12A | interleukin 12A [Source:HGNC Symbol;Acc:HGNC:5969] | 6930 | -0.074 | -0.2118 | No |
| 34 | MAP3K1 | mitogen-activated protein kinase kinase kinase 1 [Source:HGNC Symbol;Acc:HGNC:6848] | 7066 | -0.085 | -0.2181 | No |
| 35 | IFNA6 | interferon alpha 6 [Source:HGNC Symbol;Acc:HGNC:5427] | 7272 | -0.101 | -0.2292 | No |
| 36 | MAPK12 | mitogen-activated protein kinase 12 [Source:HGNC Symbol;Acc:HGNC:6874] | 8161 | -0.168 | -0.2901 | No |
| 37 | AZI2 | 5-azacytidine induced 2 [Source:HGNC Symbol;Acc:HGNC:24002] | 8242 | -0.174 | -0.2878 | No |
| 38 | TRAF2 | TNF receptor associated factor 2 [Source:HGNC Symbol;Acc:HGNC:12032] | 8322 | -0.180 | -0.2851 | No |
| 39 | IFNA7 | interferon alpha 7 [Source:HGNC Symbol;Acc:HGNC:5428] | 8533 | -0.196 | -0.2918 | No |
| 40 | IFNA1 | interferon alpha 1 [Source:HGNC Symbol;Acc:HGNC:5417] | 8640 | -0.204 | -0.2900 | No |
| 41 | MAPK8 | mitogen-activated protein kinase 8 [Source:HGNC Symbol;Acc:HGNC:6881] | 8956 | -0.228 | -0.3033 | No |
| 42 | CASP10 | caspase 10 [Source:HGNC Symbol;Acc:HGNC:1500] | 9404 | -0.264 | -0.3252 | No |
| 43 | IKBKE | inhibitor of nuclear factor kappa B kinase subunit epsilon [Source:HGNC Symbol;Acc:HGNC:14552] | 9646 | -0.287 | -0.3298 | No |
| 44 | MAPK10 | mitogen-activated protein kinase 10 [Source:HGNC Symbol;Acc:HGNC:6872] | 9699 | -0.291 | -0.3195 | No |
| 45 | ATG12 | autophagy related 12 [Source:HGNC Symbol;Acc:HGNC:588] | 9776 | -0.299 | -0.3108 | No |
| 46 | CASP8 | caspase 8 [Source:HGNC Symbol;Acc:HGNC:1509] | 10442 | -0.366 | -0.3446 | No |
| 47 | IFNB1 | interferon beta 1 [Source:HGNC Symbol;Acc:HGNC:5434] | 10801 | -0.406 | -0.3525 | Yes |
| 48 | IFNW1 | interferon omega 1 [Source:HGNC Symbol;Acc:HGNC:5448] | 10816 | -0.407 | -0.3336 | Yes |
| 49 | IRF3 | interferon regulatory factor 3 [Source:HGNC Symbol;Acc:HGNC:6118] | 10879 | -0.414 | -0.3181 | Yes |
| 50 | IKBKG | inhibitor of nuclear factor kappa B kinase regulatory subunit gamma [Source:HGNC Symbol;Acc:HGNC:5961] | 10986 | -0.428 | -0.3053 | Yes |
| 51 | NFKBIB | NFKB inhibitor beta [Source:HGNC Symbol;Acc:HGNC:7798] | 11277 | -0.468 | -0.3049 | Yes |
| 52 | TBK1 | TANK binding kinase 1 [Source:HGNC Symbol;Acc:HGNC:11584] | 11326 | -0.474 | -0.2853 | Yes |
| 53 | MAPK11 | mitogen-activated protein kinase 11 [Source:HGNC Symbol;Acc:HGNC:6873] | 11501 | -0.501 | -0.2742 | Yes |
| 54 | CHUK | component of inhibitor of nuclear factor kappa B kinase complex [Source:HGNC Symbol;Acc:HGNC:1974] | 11624 | -0.521 | -0.2581 | Yes |
| 55 | TRIM25 | tripartite motif containing 25 [Source:HGNC Symbol;Acc:HGNC:12932] | 11834 | -0.556 | -0.2470 | Yes |
| 56 | RIPK1 | receptor interacting serine/threonine kinase 1 [Source:HGNC Symbol;Acc:HGNC:10019] | 11953 | -0.584 | -0.2275 | Yes |
| 57 | CXCL8 | C-X-C motif chemokine ligand 8 [Source:HGNC Symbol;Acc:HGNC:6025] | 12297 | -0.676 | -0.2210 | Yes |
| 58 | TBKBP1 | TBK1 binding protein 1 [Source:HGNC Symbol;Acc:HGNC:30140] | 12462 | -0.730 | -0.1979 | Yes |
| 59 | MAVS | mitochondrial antiviral signaling protein [Source:HGNC Symbol;Acc:HGNC:29233] | 12646 | -0.822 | -0.1717 | Yes |
| 60 | DDX3Y | DEAD-box helicase 3 Y-linked [Source:HGNC Symbol;Acc:HGNC:2699] | 12728 | -0.890 | -0.1342 | Yes |
| 61 | DDX3X | DEAD-box helicase 3 X-linked [Source:HGNC Symbol;Acc:HGNC:2745] | 12788 | -0.949 | -0.0921 | Yes |
| 62 | NFKBIA | NFKB inhibitor alpha [Source:HGNC Symbol;Acc:HGNC:7797] | 12817 | -1.001 | -0.0450 | Yes |
| 63 | SIKE1 | suppressor of IKBKE 1 [Source:HGNC Symbol;Acc:HGNC:26119] | 12843 | -1.046 | 0.0044 | Yes |
Table: GSEA details [plain text format]

  

Fig 2: KEGG\_RIG\_I\_LIKE\_RECEPTOR\_SIGNALING\_PATHWAY      
 Blue-Pink O' Gram in the Space of the Analyzed GeneSet

  

Fig 3: KEGG\_RIG\_I\_LIKE\_RECEPTOR\_SIGNALING\_PATHWAY: Random ES distribution      
 Gene set null distribution of ES for **KEGG\_RIG\_I\_LIKE\_RECEPTOR\_SIGNALING\_PATHWAY**
